# Rural households at risk of malaria did not own sufficient insecticide treated nets at Dabat HDSS site: evidence from a cross sectional re-census

**DOI:** 10.1186/s12889-017-4906-3

**Published:** 2017-11-21

**Authors:** Kindie Fentahun Muchie, Kassahun Alemu, Amare Tariku, Adino Tesfahun Tsegaye, Solomon Mekonnen Abebe, Mezgebu Yitayal, Tadesse Awoke, Gashaw Andargie Biks

**Affiliations:** 1 Department of Epidemiology and Biostatistics, Institute of Public Health, College of Medicine and Health Sciences, University of Gondar, Gondar, Ethiopia; 2 Departement of Human Nutrition, Institute of Public Health, College of Medicine and Health Sciences, University of Gondar, Gondar, Ethiopia; 3 Departement of Health Service Management and Economics, Institute of Public Health, College of Medicine and Health Sciences, University of Gondar, Gondar, Ethiopia; 4 Dabat Research Centre Health and Demographic Surveillance System, Institute of Public Health, College of Medicine and Health Sciences, University of Gondar, Gondar, Ethiopia

**Keywords:** Altitude, Determinants, ITN, Ownership, Utilization, Dabat, HDSS, Northwest Ethiopia

## Abstract

**Background:**

Malaria is the leading cause of disease burden across the world, especially in African countries. Ethiopia has designed a five year (2011–2015) plan to cover 100% of the households in malarious areas with one insecticide treated net (ITN) for every two persons, and to raise consistent ITN utilization to at least 80%. However, evidence on ownership of ITN among malarious rural households in northwest Ethiopia is quite limited. Hence, the present study aimed at assessing ownership of ITN and associated factors among rural households at risk of malaria at Dabat Health and Demographic Surveillance System site, northwest Ethiopia.

**Methods:**

A cross sectional re-census was carried out in Dabat Health and Demographic Surveillance System site during peak malaria seasons from October to December, 2014. Data for 15,088 households at Dabat Health and Demographic Surveillance System site were used for the analysis. Descriptive measures and binary logistic regression were carried out.

**Results:**

Among those who owned at least one ITN, 53.4% were living at an altitude >2500 m above sea level. However, out of households living at an altitude <2000 m above sea level, 15.8% (95% CI 14.4%, 17.3%) owned ITN at an average of 4.3 ± 2.1 persons per ITN. Of these, 69.5% (95% CI 64.7%, 74.1%) used the ITN. Among utilizing households at malarious areas, 23.7% prioritized pregnant women and 31.4% children to use ITN. The availability of radio receiver/mobile (AOR 1.60, 95%CI 1.08, 2.35) and secondary/above educational status of household member (AOR 1.54, 95%CI 1.19, 2.04) were predictors of ownership of ITN.

**Conclusion:**

Rural households at risk of malaria did not own a sufficient number of ITN though the utilization is promising. Moreover, prioritizing children and pregnant women to sleep under ITN remains public health problems. Programmers, partners and implementers should consider tailored intervention strategy stratified by altitude in distributing ITN. ITN distribution should also be accompanied by using exhaustive promotion strategies that consider people without access to any source of information, and educating households to prioritize pregnant and under five children to sleep under ITN.

## Background

Malaria is the leading cause of disease burden across the world, especially in African countries. Globally, there were 214 million new cases of malaria in 2015 [1], of which 88% was from the African region. In the same year, there were an estimated 438,000 malaria deaths worldwide. But most of these deaths (90%) occurred in the African region [1]. Malaria is a major public health problem in Ethiopia where it is among the ten top leading causes of morbidity and mortality in children under-5 years [2, 3].

Malaria transmission exhibits a seasonal pattern in Ethiopia. The major transmission season in the country is from September to December, following the main rainy season (June/July to September), and between May and July following the end of the dry season [4]. The transmission also varies with altitude and rainfall [4–6]. In the country, areas below 2000 m above sea level (masl) were considered malarious [7]. However, malaria prevalence among individuals living at areas >2000 and > = 2500 masl were zero [8]. Accordingly, about 75% of the areas in the country are malarious and targeted to receive key control intervention [9].

Vector control is the main way to prevent and reduce malaria transmission. Insecticide-treated nets (ITNs) are effective control mechanism in a wide range of circumstances [1]. Ethiopia has designed a five year (2011–2015) plan to cover 100% of households (HHs) in malarious areas with one ITN for every two persons, and to reach at least 80% consistent ITN utilization to fight the vector [4].

ITNs as a tool for malaria control can present challenges, such as coverage and proper use [10]. Ownership and utilization of ITNs are two important indicators of monitoring progress towards the target to control the vector [11]. Ownership is important to assess the effectiveness of the distribution channels of ITNs and suggest program modifications where there are lapses [11]. However, utilization is an indicator that generates the epidemiological impact [12].

Periodic household (HH) surveys were recommended by the World Health Organization (WHO) to assess whether people at risk receive sufficient ITNs and whether there is proper use of ITNs [13]. Some evidences showed that the existence of rapid increases in ITN coverage in some of the poorest countries in Africa, but coverage remains low in large populations at risk [14]. Different studies conducted in the country and parts of the world have shown that there were a lot of ups and downs regarding ITNs ownership and utilization at HH levels [15–22]. Besides, though designing tailored intervention for the stratification by altitude within areas in Ethiopia is recommended [8], studies assessing ITN distribution by altitude were limited in the study area.

Evidences extracted from different studies have shown that ownership and utilization status of ITNs were affected by different factors including geographical, individual, HH, and environmental. Some of them were residence, literacy level of HH members, awareness about ITNs, geographical settlements of HHs, awareness on malaria prevention, sex of HH head, HH head income, availability of radio receiver, duration since ITNs were received by the HH, family size of the HH, and occupational status of head of the HH [15, 17–27]. Most studies currently emphasized on the factors affecting utilization of ITN [17, 20, 22]. However, there is an evidence in Ethiopia showing low ownership of ITNs while utilization is promising [28]. Besides, in countries, including Ethiopia, with limited resource and varying malaria transmission pattern it might be important to prioritize ownership of ITN and its factors.

Evidence about ownership of sufficient ITNs among HH in rural malarious areas, northwest Ethiopian is quite limited. Hence, the present study aimed at assessing ITN ownership and associated factors among rural HHs at risk of malaria at Dabat Health and Demographic Surveillance System (HDSS) site, northwest Ethiopia. The finding of this analysis would help to evaluate the current malaria control activities by ITN in the study setup. If properly utilized, this information will urge the decision makers to strengthen malaria control interventions effectively and efficiently.

## Methods

### Study setting

**Fig. 1:**
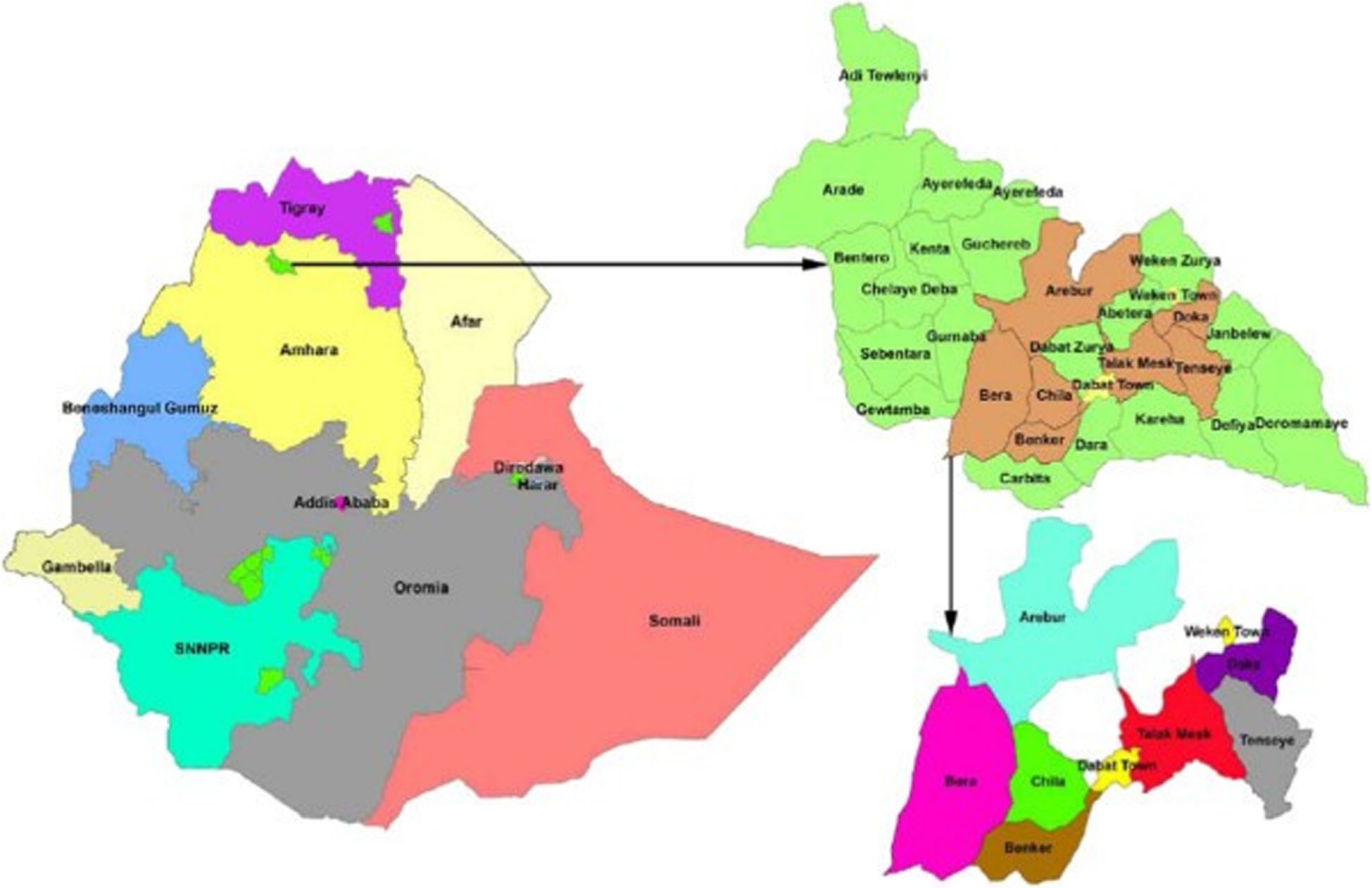
Dabat Health and Demographic Surveillance System Site in northwest Ethiopia. Kebeles in the site showed by different colors

The Dabat HDSS site is located in a rural part of the Amhara Regional State in northwest Ethiopia (Fig. 1). The altitude ranges from about 1000 to 3000 masl. Dabat district was initially selected purposively as a surveillance site for its unique three climatic conditions, namely Dega (highland and cold), Woina-dega (midland and temperate) and Kolla (lowland and hot). The choice was made with the assumption that there would be differences in morbidity and mortality in the different climatic areas. Accordingly, thirteen kebeles (nine rural and four urban) were selected after stratification of the kebeles by climatic zone. “Kebeles” are the smallest administrative units in towns and cities in Ethiopia. Dabat HDSS is a full member of the International Network of Demographic Evaluation of Populations and Their Health (INDEPTH). The detailed data collection system, data quality control, the database, and the study setting of Dabat HDSS are described at the website of the University of Gondar [29].

### Study design and population

Since the establishment of the Dabat HDSS site in November 1996, information on vital events has been being collected every six months and verbal autopsy (VA) as soon as the events happened. The cross sectional re-census was carried out at HH levels of the site in the peak malaria seasons of October to December 2014, was used for this study.

### Methods of data collection

All HHs in the nine rural and four urban kebeles were covered during the data collection period. This was done using a standardized WHO questionnaire that elicits general information about HHs, the demographic characteristics of HH members, housing and environmental conditions in the site [30]. For this analysis, the HH questionnaire which was adapted and translated to Amharic, the National language was used. Furthermore, the respondents were asked about presence, number, and condition of mosquito nets (verified by observation for hole size referencing ‘torch battery’ size D cell, diameter 33 mm, for hole size reference); and nets used the previous night. Altitude and location of each HH were recorded using the Global Positioning System (GPS).

### Variables of the study

The outcome variable considered in this study was ownership of ITNs at a HH level. In this study, all insecticide-treated nets, whether long lasting or retreated are referred to as ITNs. Ownership of ITN was defined as “whether a HH own at least one functional ITN”. With respect to ITNs utilization, a HH was taken as “utilized” if the ITNs were used in the night preceding the survey among HHs who owned them.

### Data management and analysis

Data were entered into the database, using the software household registration system (HRS) version 2.1, and exported to Stata 14.0 for further analysis. Households were divided into socioeconomic quantiles based on their scores. In order to capture wealth differences between urban and rural residences, the Principal Component Analysis (PCA) scores were generated for the two areas (urban and rural HH wealth indices) separately. The dimension of the PCA explaining 69% was taken as the score for the HH. Finally, the common factor scores were summed and ranked into lowest, lower, middle, higher, and highest. Data from the 15,088 HHs living in the Dabat HDSS site were used for exploring the distribution of ITN ownership by altitude., Out of 2502 HHs living in malarious areas (altitude <2000 masl), 2495 with complete data (associated with ITN ownership) were used for further multivariable analysis.

Descriptive measures were used to present characteristics of the HHs in the study area. Bi-variable binary logistic regression analysis was done to determine the association of the response variable and explanatory factors considered in this study. Significant variables (*p*-value < 0.25) observed in bi-variable analysis were subsequently included in multivariable binary logistic regression analysis. Finally, results were reported as statistically significant whenever *p*-values were less than 0.05. Adjusted odds ratio (AOR) was used to report the strength of association between the outcome and explanatory variables.

## Results

### Distribution of HH and ownership of ITN by altitude at Dabat HDSS site

In Dabat HDSS site the majority of HHs, 68.9%, were living at an altitude of >2500 masl, whereas 16.6% were at an altitude of <2000 masl (Table 1).

**Table 1:** Count (%) of households that own ITN by altitude at Dabat HDSS site, northwest Ethiopia, Oct. to Dec. 2014

		Altitude (in meters above sea level)				
		<2000	2000–2299	2300–2499	>2500	Total
Own ITN	Yes	396(22.5)	83 (4.7)	340(19.4)	938(53.4)	1757(11.6)
	No	2106(15.8)	265(2.0)	1497(11.2)	9463(71.0)	13,331(88.4)
Total		2502(16.6)	348(2.3)	1837(12.2)	10,401(68.9)	15,088

**Fig. 2:**
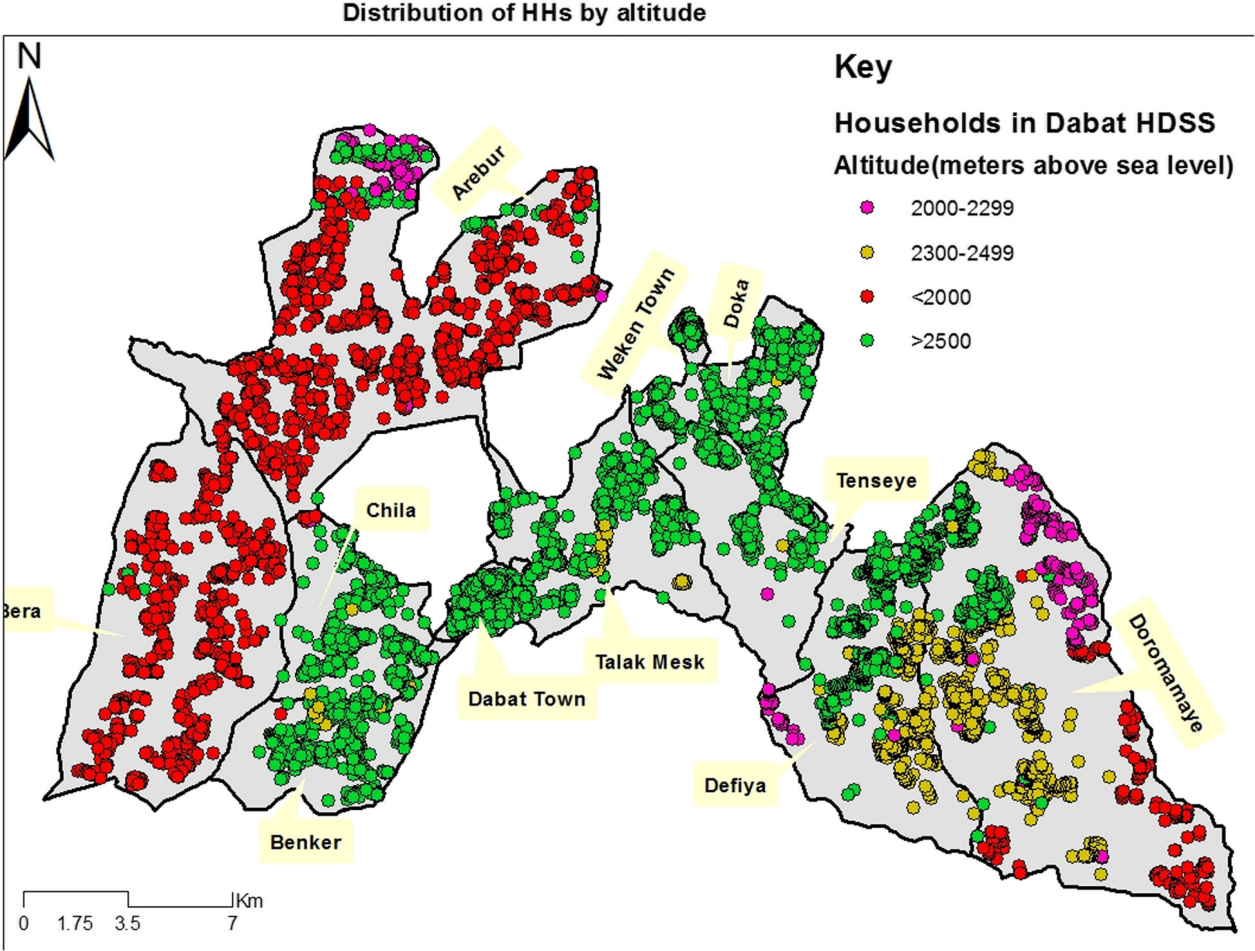
Distribution of HHs by altitude at Dabat HDSS site, northwest Ethiopia, Oct.-Dec. 2014

Moreover, almost all of the HHs in Bera and Arebur kebeles lived at <2000 masl (Fig. 2). However, the majority of the HHs in Benker, Dabat town, Talak Mesk, Chila, Weken town, Doka and Tenseye kebeles lived at >2500 masl.

Out of the HHs in the Dabat HDSS site, 11.6% owned at least one ITN (Table 1). Out of those who owned at least one ITN, 53.4%, 22.5%, 19.4%, and 4.7% lived at an altitudes of >2500 masl, <2000 masl, 2300–2500 masl, and 2000–2300 masl, respectively.

**Fig. 3:**
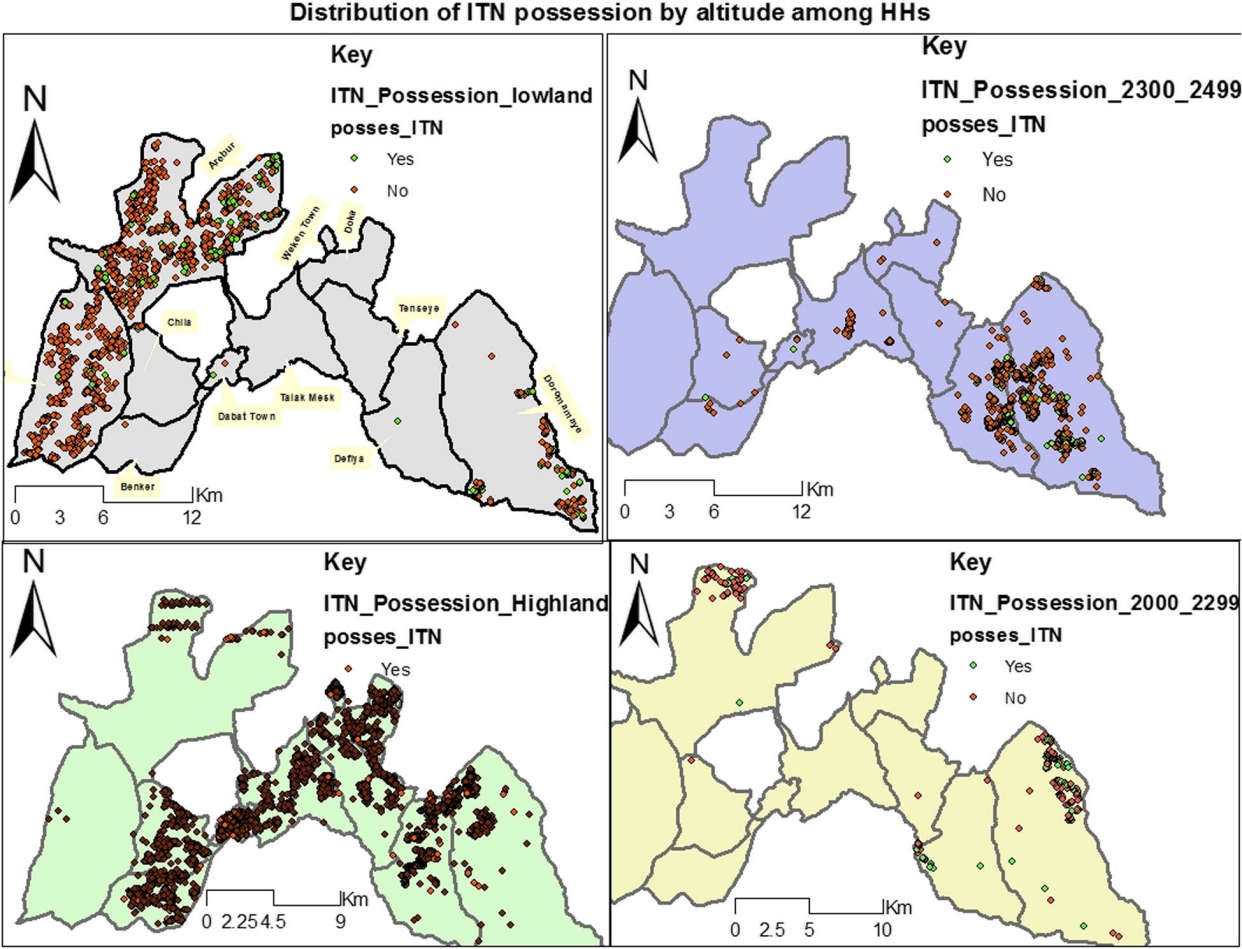
Distribution of ITN ownership by altitude among HHs at Dabat HDSS site, northwest Ethiopia, Oct.- Dec. 2014

It has been observed that some HHs living at >2500 masl owned ITN even though a large number of those living at <2000 masl did not (Fig. 3).

### Socio-demographic characteristics at malarious areas of Dabat HDSS site

Almost all, 99.9% HHs at malarious areas of Dabat HDSS site were rural residents. Regarding family size, the average size was 5.1(S.D = 2.2). With respect to HH head characteristics, 19.5% of HHs were female headed, while the majority of HH heads, 78.2% were cohabiting and 84.6% were farmers. Of the HHs at the malarious areas of the Dabat HDSS site, 17.3% owned sources of information (radio receivers or mobile phones) (Table 2).

**Table 2:** Household socio-demographic characteristics at malarious areas of Dabat HDSS site, northwest Ethiopia, Oct. to Dec. 2014

Variables	Frequency	Percent
HH access to source information		
No	2063	82.7
Yes	432	17.3
Sex of HH head		
Female	486	19.5
Male	2009	80.5
Educational level in HH		
Un educated	866	34.7
Primary	1391	55.81
Secondary and above	238	9.5
HH size		
1–4	1034	41.4
5+	1461	58.6
HH head Age in years		
14–32	625	25.1
33–44	658	26.4
45–56	630	25.3
57–96	581	23.3
HH head marital status		
Cohabit^a^	1950	78.2
Not cohabit^b^	544	21.8
Wealth status		
Low income	346	14.9
Middle income	913	39.3
Better income	1063	45.8
Place of residence		
Rural	2493	99.9
Urban	2	0.1
Farming main occupation of head		
Yes	2112	84.6
No^c^	383	15.4

^a^Married or living together

^b^Single, divorced, separated or widowed

^c^student, Employee, shepherd, house worker; Private, merchant, unemployed, retired, disabled, housewife

### Ownership and utilization of ITN at malarious areas of Dabat HDSS site

Only 15.8% (95%CI: 14.4%, 17.3%) of the HHs at malarious areas of Dabat HDSS site had at least one ITN with an average of 4.3 (S.D = 2.1) persons per ITN. Out of these, 69.5% (95%CI: 64.7%, 74.1%) utilized the net. Of the utilizing HHs, only 31.4% prioritized children and 23.7% pregnant women to sleep under ITNs (Table 3).

**Table 3:** Ownership and utilization of ITNs among household at malarious areas of Dabat HDSS site, northwest Ethiopia, Oct. to Dec. 2014

Characteristics	Frequency	Percent
ITN ownership		
Yes	394	15.8
No	2101	84.2
Utilization of ITNs last night		
Yes	274	69.5
No	120	30.5
Priority to sleep under ITN		
All equal	240	87.6
Pregnant women	65	23.7
Child	86	31.4
Aged person	26	9.5
Others^a^	40	14.6

^a^husband & wife, mother & child, myself, patient, women

### Determinants of ITNs ownership at malarious areas of Dabat HDSS site

In the bi-variable binary logistic regression analysis, variables including access to information source, maximum educational status, family size and age of HHs head were significantly associated at 0.25 level of significance. Consequently, all these were considered for the final model. Possession of information source and maximum educational status HH members were statistically significant determinants of ownership of ITN at malarious areas at the Dabat HDSS site, northwest Ethiopia, at 5% level of significance.

HH members with highest educational status (secondary and above) were 60% (AOR = 1.60; *p*-value = 0.018; 95%CI: 1.08, 2.35) more likely to own ITNs as compared to completely uneducated HH members. A household possessing some source of information, either radio receiver or mobile phone was 54% (AOR = 1.54; p-value = 0.001; 95%CI: 1.19, 2.04) more likely to have ITNs as compared to those who didn’t have any of the possible information sources (Table 4).

**Table 4:** Multivariable analysis for determinants of ownership of ITN at malarious areas at Dabat HDSS site, northwest Ethiopia, Oct. to Dec. 2014

Variables	ITN ownership		COR[95% CI]	AOR [95% CI]
	Yes	No		
Access to info source				
Yes	97(22. 5%)	335(77.5%)	1.72[1.33, 2.23]	1.54[1.19, 2.04]
No	297(14.4%)	1766(85.6%)	1	1
HH max. Educational status				
Un educated	117(13.5%)	749(86.5%)	1	1
primary	226(16.2%)	1165(83.8%)	1.24[0.98, 1.58]	1.19[0.92, 1.55]
secondary and above	51(21.4%)	187(78.6%)	1.74[1.21, 2.52]	1.60[1.08, 2.35]
HH family size				
1–4	151(14.6%)	883(85.4%)	1	1
5+	243(16.6%)	1218(83.4%)	1.17[0.94, 1.45]	1.10[0.85, 1.42]
HH head Age(years)				
14–32	107(17.1%)	518(82.9%)	1	1
33–44	119(18.1%)	539(81.9%)	1.07[0.80, 1.42]	1.02[0.74, 1.40]
45–56	88(14.0%)	542(86.0%)	0.79[0.58, 1.07]	0.72[0.51, 1.00]
57–96	80(13.8%)	501(86.2%)	0.77[0.56, 1.06]	0.78[0.57, 1.08]

## Discussion

This study examined ownership and utilization of ITN that enables us to compare with the national target in Ethiopia. It has been identified that housing access to some sources of information as well as maximum higher educational status attainment in HHs contribute to ITN ownership at the malarious areas of Dabat HDSS site.

According to the result of this study the proportion of ITN ownership is low, which remained too far from the 2015 national target of universal coverage. That is, less than one-fifth of the studied HHs at malarious areas had at least one ITN with two fold of the expected persons per ITN. It has been noted that HHs in Ethiopia are unevenly distributed at various altitudes within many districts and kebeles [31]. However, it has been observed that a large percentage of ITN ownership was found at malaria free areas of the Dabat HDSS site. This shows that ITN distribution might not consider altitude to prioritize HHs at malarious areas of the site. It may also show us wastage of resources in that there is unnecessary distribution of ITNs to those who did not deserve to have. Such kind of distribution of ITNs could be due to common barriers to delivery including cost, stock-outs and poor logistics [32]. The distribution of ITNs should better be accompanied by tailored intervention strategy stratified by altitude [33].

Ownership of ITN at malarious areas of the Dabat DHSS seems lower than that of most study results in Ethiopia and elsewhere. For instance, reports show 81.7% in Itang [22], 49.8% in southern Nigeria [34], 65.5% in Kersa [20], 62.4% in Gursum [18], and 58.8% in Arbaminch Zuria [15]. This may be due to a low distribution and/or retention of nets at the malarious areas of Dabat HDSS, northwest Ethiopia. However, nearly three-quarters of the ITN owner HHs used at least one of their ITNs in the night preceding the survey, showing the utilization of ITNs in the study site is promising as far as the 2015 national target of 80% utilization. A similar study in southern Nigeria indicates a 75.4% utilization [34]. Another study in Itang, Gambella Region, Ethiopia, also shows a lower proportion of HHs (52.3%) used ITNs in the night preceding the survey [22]. However, small proportion of HH at malarrious areas of Dabat HDSS site prioritize vulnerable groups including pregnant women and under five children to sleep under ITN. In distributing ITNs, educating HHs to prioritize pregnant and under five children to sleep under ITN should be considered [33].

A HH with some source of information, including radio receivers or mobile phones are more likely to have ITNs. This may be due to HHs with respondents that knew the cause of malaria, and have heard about ITN are more likely to own ITNs than their counterparts [18]. A related study in central Ethiopia consistently shows that housing access to some source of information contributes to the ownership of ITN [33]. Here, the Government needs to consider ITN promotion strategies targeting HHs with no access of information [33].

Households with a maximum educational status (secondary and above) are more likely to have ITNs as compared to those with completely uneducated members in the HHs. This may imply, the existence of a member with advanced educational status would lead the HH to be keen enough to collect their share from the health posts and avail themselves of the dates and places of distribution. It may also imply that collected ITNs in HHs with a better educated member would not wear out fast and get lost because of improper handling. This is attributable to knowledge about the sources of ITN, and belief that ITN does not protect or is not important to prevent malaria [35]. Consistently, some studies show participants with higher educational status improves ownership of ITN [28, 36]. This is due to the fact that those with higher educational level may have knowledge about ITNs, malaria transmission, and mosquitoes in their school stay tending them to acquire the ITN [28, 36]. In contrast, another study shows that HHs with no formal education own ITN above 35-fold more compared to those with formal education [35]. This difference could be attributable to the presence of a highly educated HH member capable of fostering ownership of ITN, no matter how un educated the head is.

As a limitation this work did not consider the rainfall and could not identify why ownership of ITN is greater at very high altitudes where malaria transmission is very rare. Further, this analysis was unable to establish causality due to cross sectional nature of the design and use of logistic regression. More factors including number of beds, draining/refilling of mosquito breeding sites, presence of rivers or streams, distance from rivers or streams, distance from health services, and access of vehicle transportation that could affect ownership of ITN were not included. So, the results should be considered with this limitations in mind.

## Conclusion

In this study, the rural HH at malarious areas did not own sufficient ITNs, whereas overall utilization was promising. Moreover, prioritizing vulnerable groups (children and pregnant women) to sleep under ITN remains a public health concern in the study area. Programmers, partners, and implementers should consider tailored intervention strategy stratified by altitude in distributing ITN to achieve malaria eradication in the country. Additionally, ITN distribution should be accompanied by using exhaustive promotion strategies that consider people without access to any source of information, and educating HHs to prioritize key population (pregnant and under five children) to sleep under ITN.

## Abbreviations

AOR: Adjusted odds ratio
COR: Crude odds ratio
GPS: Global Positioning System
HDSS: Health and demographic surveillance system
HH: Household
HHs: Households
HRS: Household registration system
INDEPTH: International Network of Demographic Evaluation of Populations and Their Health
ITN: Insecticide treated net
ITNs: Insecticide treated nets
Masl: Meters above sea level
OR: Odd ratio
PCA: Principal Component Analysis
S.D.: standard deviation
TV: Television
VA: Verbal autopsy
